# The Fate of the Physician

**Published:** 1847-10

**Authors:** 


17. *The Fate of the Physician.*—"Another physician, Dr. D. B. Hall, died yesterday—this is the fourth."—*New Orleans Paper.*

Such are the brief, cold terms in which the public are told that Medicine is offering up victim after victim, on the alter of professional duty.

Where are now the Hydropaths, Homœopaths, Root Doctors, and the whole legion of quacks? They are silent —they have probably fled, to seek in some place of safety for dupes and victims. And where are now the flippant sneerers at the uncertainty of medical science—"the Doctors' quarrels"—"the Doctors' bills"—"the Doctors' rapacity?" Silent all! no voice is heard to breathe a word of reproach or ridicule. No! no! the talk now is, "Our physicians are laboring, dying? Such is ever the fate of Medicine and medical men. In the hour of suffering, or of danger, they are sought out with eager zeal and rewarded with garrulous gratitude; but let that hour pass, and the danger, and he whose skill averted it—the suffering, and he whose toil made it tolerable—are alike forgotten, and the public turn from their long-tried physician, and give the reward which he has so dearly earned, to the ignorance, the impudence of the nostrum-vender, or the new-system-men.

And what is our duty when thus treated? *Go onward! Look upward!* Go onward! the path of duty is before you. Look upward! the reward is on high.—*Ibid.*

